# *TCERG1L* allelic variation is associated with cisplatin-induced hearing loss in childhood cancer, *a PanCareLIFE study*

**DOI:** 10.1038/s41698-021-00178-z

**Published:** 2021-07-14

**Authors:** A. J. M. Meijer, F. A. Diepstraten, T. Langer, L. Broer, I. K. Domingo, E. Clemens, A. G. Uitterlinden, A. C. H. de Vries, M. van Grotel, W. P. Vermeij, R. A. Ozinga, H. Binder, J. Byrne, E. van Dulmen-den Broeder, M. L. Garrè, D. Grabow, P. Kaatsch, M. Kaiser, L. Kenborg, J. F. Winther, C. Rechnitzer, H. Hasle, T. Kepak, K. Kepakova, W. J. E. Tissing, A. L. F. van der Kooi, L. C. M. Kremer, J. Kruseova, S. M. F. Pluijm, C. E. Kuehni, H. J. H. van der Pal, R. Parfitt, C. Spix, A. Tillmanns, D. Deuster, P. Matulat, G. Calaminus, A. E. Hoetink, S. Elsner, J. Gebauer, R. Haupt, H. Lackner, C. Blattmann, S. J. C. M. M. Neggers, S. R. Rassekh, G. E. B. Wright, B. Brooks, A. P. Nagtegaal, B. I. Drögemöller, C. J. D. Ross, A. P. Bhavsar, A. G. am Zehnhoff-Dinnesen, B. C. Carleton, O. Zolk, M. M. van den Heuvel-Eibrink, the PanCareLIFE Consortium, A. C. H. de Vries, M. van Grotel, E. van Dulmen-den Broeder, A. L. F. van der Kooi, L. C. M. Kremer, H. J. H. van der Pal, G. Calaminus, A. E. Hoetink, M. M. van den Heuvel-Eibrink, and the CPNDS Consortium

**Affiliations:** 1 Princess Máxima Center for Pediatric Oncology, Utrecht, The Netherlands; 2 Department of Pediatric Oncology and Hematology, University Hospital for Children and Adolescents, Lübeck, Germany; 3 Department of Internal Medicine, Erasmus Medical Center, Rotterdam, The Netherlands; 4 Department of Medical Microbiology and Immunology, University of Alberta, Edmonton, AB Canada; 5 Department of Pediatric Oncology, Erasmus MC – Sophia Children’s Hospital, Rotterdam, The Netherlands; 6 Oncode Institute, Utrecht, The Netherlands; 7 German Childhood Cancer Registry, Institute of Medical Biostatistics, Epidemiology and Informatics, University Medical Center of the Johannes Gutenberg University Mainz, Mainz, Germany; 8 Institute of Medical Biometry and Statistics, Faculty of Medicine and Medical Center, University of Freiburg, Freiburg, Germany; 9 Boyne Research Institute, Drogheda, Ireland; 10 VU Medical Center, Amsterdam, The Netherlands; 11 Department of Neurooncology, IRCCS Istituto Giannina Gaslini, Genova, Italy; 12 Childhood Cancer Research Group, Danish Cancer Society Research Center, Copenhagen, Denmark; 13 Department of Clinical Medicine, Faculty of Health, Aarhus University and University Hospital, Aarhus, Denmark; 14 Department of Pediatrics and Adolescent Medicine, Copenhagen University Hospital Rigshospitalet, Copenhagen, Denmark; 15 Department of Pediatrics, Aarhus University Hospital, Aarhus, Denmark; 16 University Hospital Brno, Brno, Czech Republic; 17 International Clinical Research Center (FNUSA-ICRC), Brno, Czech Republic; 18 Department of Pediatric Oncology, University of Groningen, University Medical Center Groningen, Groningen, The Netherlands; 19 Department of Obstetrics and Gynecology, Erasmus MC – Sophia Children’s Hospital, Rotterdam, The Netherlands; 20 Department of Pediatric Oncology, Academic Medical Center Amsterdam, Amsterdam, The Netherlands; 21 Department of Children Hemato-Oncology, Motol University Hospital Prague, Prague, Czech Republic; 22 Institute of Social and Preventive Medicine, University of Bern, Bern, Switzerland; 23 Pediatric Hematology and Oncology, University Children’s Hospital Bern, University of Bern, Bern, Switzerland; 24 Department of Phoniatrics and Pedaudiology, University Hospital Münster, Westphalian Wilhelm University, Münster, Germany; 25 Pediatric Hematology and Oncology, University Children’s Hospital Muenster, Muenster, Germany; 26 Department of Otorhinolaryngology, Head and Neck Surgery, University Hospital Utrecht, Utrecht, The Netherlands; 27 Institute of Social Medicine and Epidemiology, University of Lübeck, Lübeck, Germany; 28 Department of Internal Medicine, University Hospital of Schleswig-Holstein, Campus Lübeck, Lübeck, Germany; 29 Epidemiology and Biostatistics Unit and DOPO Clinic, IRCCS Istituto Giannina Gaslini, Genova, Italy; 30 Department of Pediatric and Adolescent Medicine, Medical University of Graz, Graz, Austria; 31 Department of Pediatric Oncology/Hematology/Immunology, Stuttgart Cancer Center, Olgahospital, Stuttgart, Germany; 32 BC Children’s Hospital Research Institute, Vancouver, BC Canada; 33 Division of Translational Therapeutics, Department of Pediatrics, University of British Columbia, Vancouver, BC Canada; 34 Audiology and Speech Pathology Department, BC Children’s Hospital, Vancouver, BC Canada; 35 Departement of Otorhinolaryngology, Erasmus Medical Center, Rotterdam, The Netherlands; 36 Faculty of Pharmaceutical Sciences, University of British Columbia, British Columbia, Canada; 37 Department of Medical Genetics, University of Alberta, Edmonton, AB Canada; 38 Institute of Clinical Pharmacology, Brandenburg Medical School, Rüdersdorf, Germany

**Keywords:** Genome-wide association studies, Paediatric cancer

## Abstract

In children with cancer, the heterogeneity in ototoxicity occurrence after similar treatment suggests a role for genetic susceptibility. Using a genome-wide association study (GWAS) approach, we identified a genetic variant in *TCERG1L* (rs893507) to be associated with hearing loss in 390 non-cranial irradiated, cisplatin-treated children with cancer. These results were replicated in two independent, similarly treated cohorts (*n* = 192 and 188, respectively) (combined cohort: *P* = 5.3 × 10^−10^, OR 3.11, 95% CI 2.2–4.5). Modulating *TCERG1L* expression in cultured human cells revealed significantly altered cellular responses to cisplatin-induced cytokine secretion and toxicity. These results contribute to insights into the genetic and pathophysiological basis of cisplatin-induced ototoxicity.

Survival probabilities for pediatric cancer have increased tremendously over the past decades^1^. Cisplatin is a highly effective chemotherapeutic agent for an important subset of childhood cancers that depend on this drug for curation. However, the occurrence of irreversible hearing loss that occurs in ~50% of cisplatin-treated children is a serious clinical challenge^2,3^. Young age at cancer diagnosis, high total cumulative dose (TCD) of cisplatin, cranial irradiation, and/or subsequent carboplatin use might increase the risk of developing hearing loss^4–6^. Understanding the biology of cisplatin-induced hearing loss and identifying risk factors that could predict ototoxicity is highly relevant as children are at a critical stage of their speech and language development, with the added risk of experiencing social, emotional, or vocational difficulties related to hearing loss. This ultimately impacts development and quality of life during treatment but also later in life^7^.

The significant heterogeneity in the occurrence of ototoxicity among similarly treated patients suggests that genetic susceptibility contributes to cisplatin-related hearing loss^8^. Therefore, we performed a GWAS in a discovery cohort of 390 cisplatin-treated, non-cranial-irradiated European children with cancer (*n* = 168 (43.0%) with hearing loss, Supplementary Table 1)^9^. This cohort was assembled by the European initiative of the PanCareLIFE (PCL) group (http://www.pancarelife.eu/)^10^. Cases were defined as having deleterious hearing loss according to Muenster ≥2b after the aforementioned treatment and were compared to subjects with Muenster 0–2a (Supplementary Table 2)^11^. A two-stage design GWAS was conducted consisting of one discovery cohort and two replication cohorts (Supplementary Fig. 1). In the discovery cohort, a logistic regression model was applied, including age at diagnosis, sex, cisplatin TCD, and principal components 1–4, with the assumption of an additive effect of the minor allele in the model (Supplementary Tables 1 and 3, and see the “Methods” section).

In the first stage, the discovery cohort (D) GWAS analysis identified eight suggestive loci (*P* < 1.0 × 10^−5^; Table 1, and Supplementary Figs. 1 and 2)^12^, which were assessed in a second stage by pursuing replication of suggestive variants in a first, independent Canadian replication cohort (R1) of non-cranial irradiated, cisplatin-treated children (*n* = 192; 115 (59.9%) with hearing loss) (Supplementary Tables 1 and 3, and Supplementary Fig. 1)^9^. Rs893507 showed evidence of replication (*P* = 0.01), resulting in a combined OR of 2.77, adjusted for age at diagnosis, sex, cisplatin TCD, and principal components 1–4 (combined analysis 1: *P* = 4.5 × 10^−7^, 95% CI 1.9–4.1; Table 1). This genetic variant is located in an intron of the Transcription Elongation Regulator 1 Like (*TCERG1L*) gene (Fig. 1, Supplementary Table 4). This variant could potentially disrupt RNA splicing, resulting in loss of exons, or in the inclusions of introns, with a subsequently altered protein expression. Next, analysis in a second independent replication cohort, consisting of PCL childhood cancer survivors (R2) including 188 non-cranial-irradiated cisplatin-treated subjects (94 (50.0%) with hearing loss), confirmed the findings observed in the first replication cohort (Supplementary Tables 1 and 3, and Supplementary Fig. 1)^9^.

**Table 1:** GWAS results in genome-wide suggestive loci in the PCL discovery cohort, Canadian first replication cohort, PCL second replication cohort, and combined analyses.

SNP	Chr	Position	Ref/Eff	Nearest gene	Dist (kb)	PCL discovery cohort (D) *N* = 390^a^			Canadian first replication cohort (R1) *N* = 192^b^			Combined analysis 1 (D & R1)			PCL second replication cohort (R2) *N* = 188^c^			Combined analysis 2 (D, R1 & R2)		
						EAF	OR (95% CI)	*P*	EAF	OR (95% CI)	*P*	OR (95% CI)	*P*	*I*^2^	EAF	OR (95% CI)	*P*	OR (95% CI)	*P*	*I*^2^
rs10928931	2	130522107	G/C	AC079776.1	104.7	0.92	5.16 (2.5–10.5)	5.67E^–^^06^	NA^d^	NA^d^	NA^d^	NA	NA	NA	NA	NA	NA	NA	NA	NA
rs62178885	2	133645476	G/A	NCKAP5	0.0	0.36	2.10 (1.5–2.9)	7.05E^–^^06^	0.33	1.06 (0.6–1.8)	0.81	1.72 (1.3–2.3)	9.67E^−05^	80.2	NA	NA	NA	NA	NA	NA
rs75426794	6	28274651	A/G	PGBD1	4.3	0.17	2.55 (1.7–3.8)	4.26E^−^^06^	0.20	0.85 (0.5–1.6)	0.61	1.84 (1.3–2.6)	3.38E^−^^04^	88.3	NA	NA	NA	NA	NA	NA
rs9498000	6	148645188	G/A	SASH1	0.0	0.07	3.80 (2.1–6.8)	7.45E^−^^06^	0.06	0.69 (0.3–1.8)	0.44	2.40 (1.5–4.0)	5.79E^−^^04^	88.6	NA	NA	NA	NA	NA	NA
rs893507	10	133013187	T/C	TCERG1L	0.0	0.15	2.66 (1.7–4.1)	9.03E^−06^	0.09	3.37 (1.3–8.9)	0.01	2.77 (1.9–4.1)	4.45E^−07^	0.0	0.11	5.45 (2.3–12.8)	1.02E^−04^	3.11 (2.2–4.5)	5.31E^−10^	8.1
rs61945410	12	125980301	C/T	TMEM132B	0.0	0.27	0.40 (0.3–0.6)	9.67E^−06^	0.22	2.27 (1.2–4.2)	0.01	0.68 (0.5–1.0)	2.73E^−02^	95.4	NA	NA	NA	NA	NA	NA
rs966556	13	20749239	C/T	PPIAP28	3.9	0.66	0.48 (0.4–0.7)	8.31E^−06^	0.70	0.83 (0.5–1.4)	0.49	0.56 (0.4–0.7)	3.17E^−05^	66.9	NA	NA	NA	NA	NA	NA
rs74032316	15	96571138	T/C	RP11-4G2.1	0.2	0.24	2.30 (1.6–3.3)	5.95E^−06^	0.24	1.05 (0.6–1.8)	0.86	1.81 (1.3–2.4)	1.10E^−04^	82.1	NA	NA	NA	NA	NA	NA

*Chr* chromosome, *CI* confidence interval, *Dist* distance, *EAF* effect allele frequency, *Eff* effect allele, *I*^2^ percentage of variants in the combined analysis that is attributable to study heterogeneity, *NA* not assessable, *OR* odds ratio, *Ref* reference allele.

^a^Imputation quality > 85%. Median age at diagnosis 11.1 years (0.0–18.8); median age at audiological testing 11.8 years (0.3–19.0); median total cumulative dose cisplatin 480 mg/m^2^ (range: 40–950 mg/m^2^). Seventy-six (19.5%) patients had been treated with additional carboplatin. One hundred sixty-eight (43.1%) patients developed Muenster ≥ 2b hearing loss.

^b^Median age at diagnosis 4.1 years (0.1–18.8); median cumulative dose cisplatin 400 mg/m^2^ (300–480 mg/m^2^). One hundred fifteen patients (59.9%) developed Muenster ≥ 2b hearing loss.

^c^Median age at diagnosis 11.1 years (0.3–18.0); median cumulative dose of cisplatin 480 mg/m^2^ (83–770). Ninety-four survivors (50.0%) developed Muenster ≥ 2b hearing loss.

^d^Not assessable as more than 5% of the individuals had a missingness threshold lower than 0.9.

**Fig. 1:**
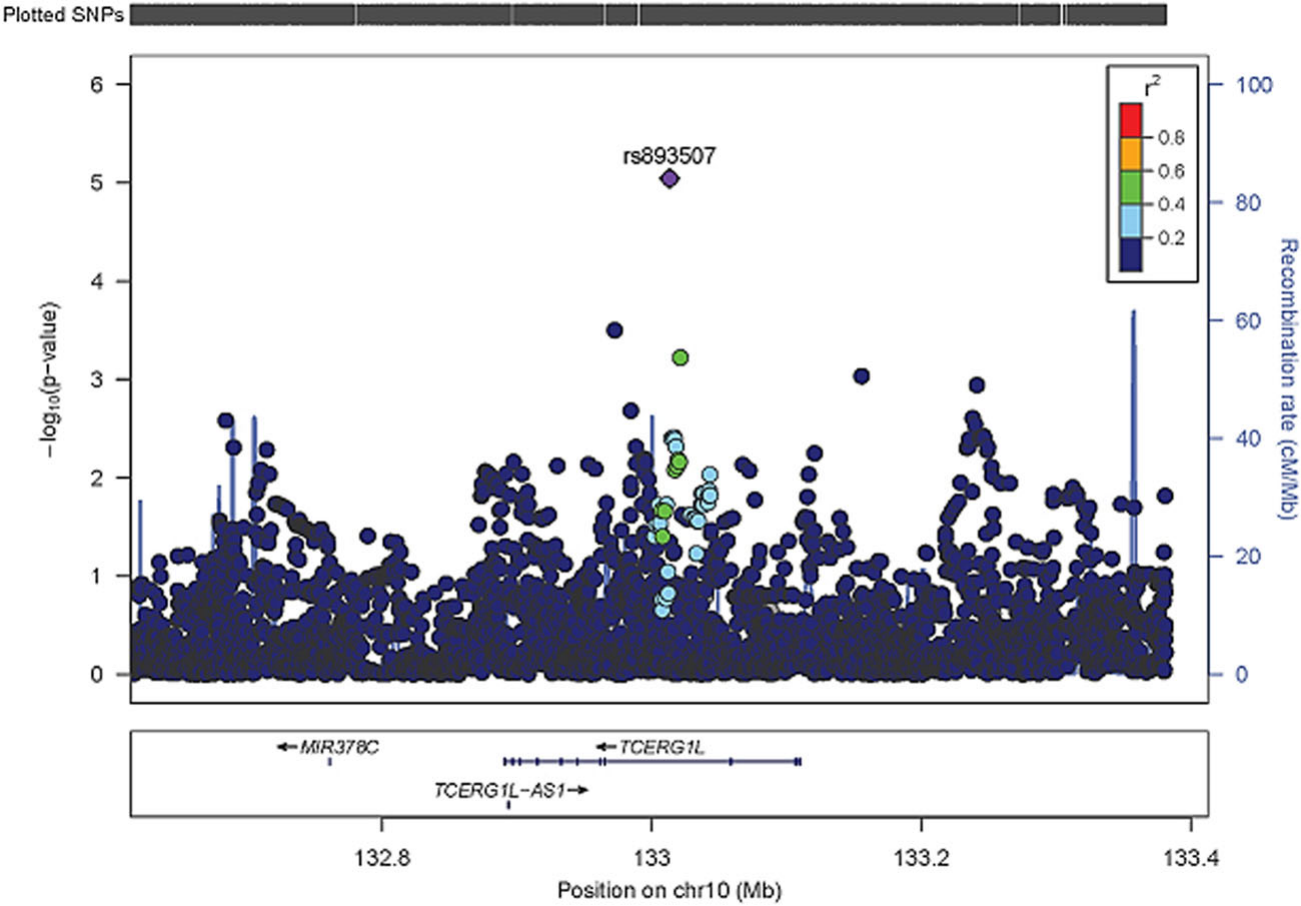
GWAS analysis uncovers variation at the *TCERG1L* locus that is associated with cisplatin-induced hearing loss. The purple diamond (genotyped SNP) is the SNP with the lowest *p*-value (*P* < 1.0 × 10^−5^) in the region. The color of the remaining SNPs represents the LD of these variants (genotyped or imputed) with the top variant. The blue lines represent recombination rates in this locus.

Combined analysis of these three cohorts represents the largest cohort to date of non-cranial irradiated, cisplatin-treated childhood cancer patients and survivors. Our results showed that the genetic variant rs893507 was associated with deleterious hearing loss at genome-wide significance. Carriership of the C-allele of this newly discovered variant increases the odds of developing serious cisplatin-induced hearing loss in children treated for cancer 3.11-fold, adjusted for age at diagnosis, sex, cisplatin TCD, and principal components (combined analysis 2: *P* = 5.3 × 10^−10^, 95% CI 2.2–4.5; Table 1; Supplementary Fig. 3).

Although a specific cisplatin threshold dose associated with ototoxicity has not been found previously^13^, an association was observed between deleterious hearing loss and cisplatin TCD continuously (OR 1.35, 95% CI 1.1–1.5) as well as after stratification (360–480 mg/m^2^: OR 2.4, 95% CI: 1.2–4.8; >480 mg/m^2^: OR 3.1, 95% CI 1.7–6.0), adjusted for age at diagnosis and sex. Next, a potential cisplatin dose–response effect with *TCERG1L* was estimated. By modeling cisplatin dose as a continuous variable, effect modification (*P* = 0.04) was observed. After stratification, a dose–response effect was neither observed for 360–480 mg/m^2^ (*P* = 0.9), nor for >480 mg/m^2^ (*P* = 0.1) as power was lost. Future studies with larger sample sizes are needed to accurately model the effect of cisplatin dose.

A possible association between the SNP and age-related hearing loss was evaluated. By contrast, rs893507 (*TCERG1L*) was not associated with age-related hearing loss in a general population (lowest *P* = 0.2 in the CHARGE cohort GWAS; Supplementary Table 5 and Supplementary Fig. 1)^14^. Similar results were reported in the Oxford PheWAS database for congenital conductive and sensorineural hearing loss (*P* = 0.09), and other hearing loss (*P* = 0.2) (http://big.stats.ox.ac.uk/variant/10:133013187-T-C). This suggests that rs893507 (*TCERG1L*) is specific for cisplatin-induced hearing loss.

The *TCERG1L* gene, a paralog of *TCERG1*, is a transcription elongation regulator that has been described to be involved in the pathogenesis of cancer and non-cancer-related diseases, but it has not previously been associated with chemotherapy-induced hearing loss. Previous studies revealed an association with inflammatory bowel disease, as well as with colon cancer predisposition suggesting that *TCERG1L* influences immunological pathways^15–17^. *TCERG1L* is expressed in the human brain, gut, thyroid, stem cells, adenoid, and tonsils, (http://biogps.org/#goto=genereport&id=256536) (https://www.genecards.org/cgi-in/carddisp. pl? gene=TCERG1)^18–20^, and more importantly also in human cochlear inner and outer hair cells^21^ as well as murine cochlear inner hair cells^20^.

Next, we performed transient *TCERG1L* silencing and overexpression experiments in vitro, to examine the effect of *TCERG1L* expression on cisplatin cytotoxicity and inflammatory response in cultured human cells. We observed that modulating *TCERG1L* expression significantly altered cell viability in response to cisplatin treatment, where *TCERG1L* overexpression and silencing, respectively, protected and sensitized cells to cisplatin toxicity. Overall, modulation of *TCERG1L* expression significantly shifted the cisplatin CC_50_ fourfold (Fig. 2a). Consistent with enhanced resistance to cisplatin, *TCERG1L* overexpression reduced pro-inflammatory IL-8 cytokine secretion in response to cisplatin treatment; whereas *TCERG1L*-silencing had the opposite effect, increasing the amount of IL-8 secreted in response to cisplatin treatment (Fig. 2b). We also found this inverse correlation between *TCERG1L* mRNA expression and pro-inflammatory cytokine (IL-6) expression in response to cisplatin treatment by RNA-seq in various mouse tissues (Fig. 2c, d). These data indicate that *TCERG1L* function contributes to the cell’s response to cisplatin exposure, which warrants further research and a comprehensive examination of the genes transcriptionally regulated by *TCERG1L*. In embryonic stem cells, *TCERG1L* expression is subject to epigenetic regulation^18^. Hence, further investigation of the interaction of this variant and epigenetic regulation is also warranted.

**Fig. 2:**
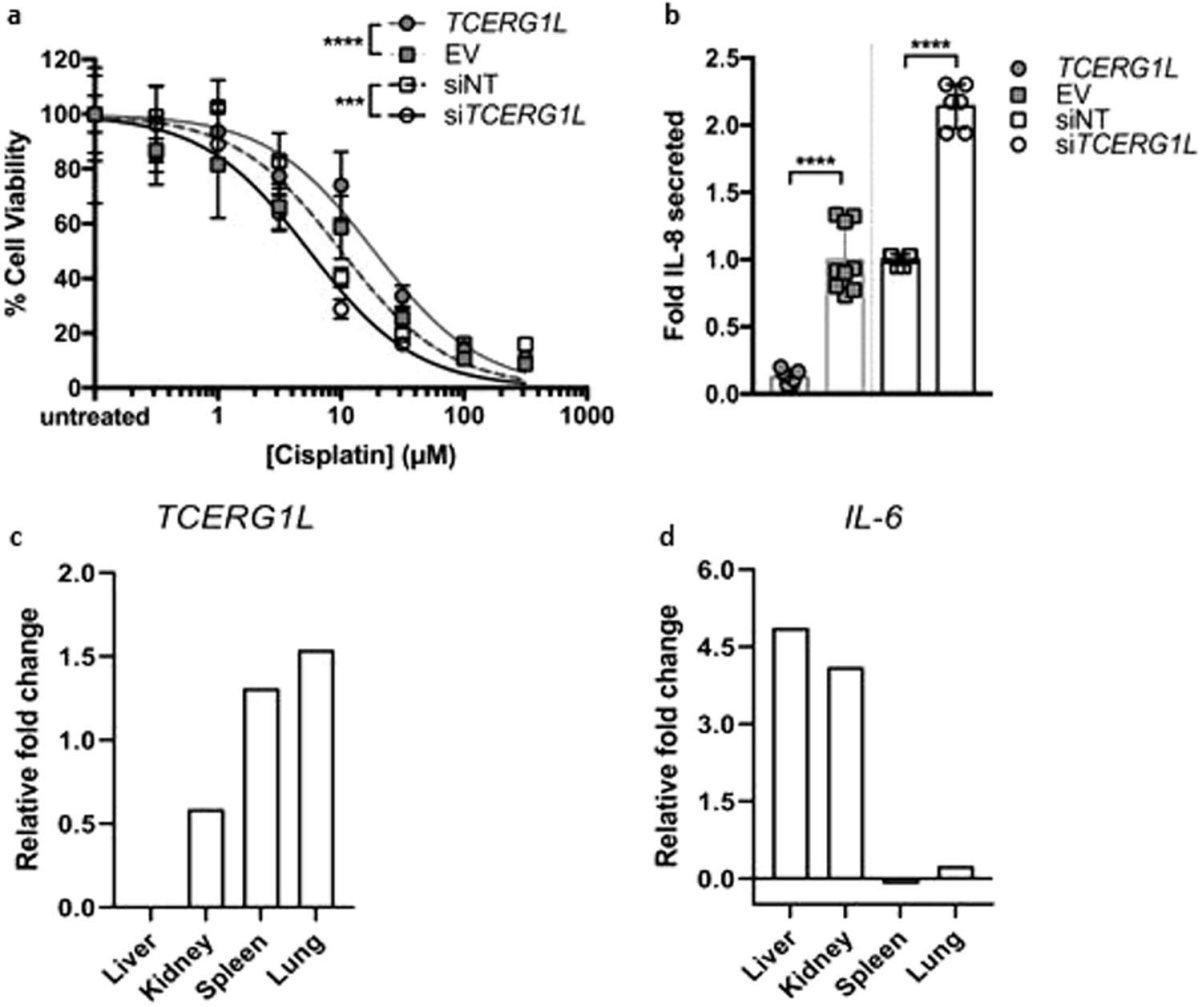
*TCERG1L* expression influences in vitro and in vivo responses to cisplatin. **a** *TCERG1L* overexpression (*TCERG1L*) and silencing (si*TCERG1L*) in HeLa cells significantly reduces and enhances cisplatin cytotoxicity, respectively, compared to empty vector (EV) or non-targeting (siNT) controls. Cisplatin CC_50_ was 5.5 μM (si*TCERG1L*) and 18.6 μM (*TCERG1L*). Control conditions (EV, siNT) cisplatin CC_50_ was 10 μM. ***P < 0.001; ****P < 0.0001 using extra sum of squares *F* test; *n* = 21 from three independent experiments (overexpression) and *n* = 9 from two independent experiments (silencing). Data are shown as the mean and standard deviation. **b** Pro-inflammatory IL**-**8 secretion at 100 μM cisplatin was significantly reduced, or enhanced, by *TCERG1L* overexpression and silencing in HeLa cells compared to their respective controls. ****P < 0.0001 using two-tailed Student *t*-test; *n* = 6 or 9 from two independent experiments. Standard deviation is shown. Overexpression and silencing experiments were performed separately but shown on the same axes for comparison in **a** and **b**. RNA expression changes of *TCERG1L* (**c**) and IL-6 (**d**) across several mouse tissues following cisplatin exposure. Relative fold changes were calculated per organ against saline-injected controls. The data set is publicly available in the NCBI Gene Expression Omnibus (GEO) under accession number GSE117167.

Notably, we selected patients in the study cohorts with bilateral hearing loss who had not received cranial irradiation, anticipating the fact that cranial radiotherapy is a dominant treatment component that may override the effect of genetic susceptibility of cisplatin-induced ototoxicity. This effect was illustrated by the fact that rs893507 (*TCERG1L*) carriership was associated with cisplatin-induced hearing loss in non-cranial-irradiated, cisplatin exposed childhood cancer survivors of the PCL second replication cohort but that the significance was lost after 553 cranial-irradiated subjects were added to the analyses (Supplementary Table 6).

In this work, we report a genetic variant associated with cisplatin-induced hearing loss stemming from childhood cancer treatment. Our genetic discovery showed genome-wide significance upon further study in additional patient cohorts including two independent international cohorts. In total, our study included 770 patients which is a relatively large cohort considering that childhood cancer is very rare. The strength of association for rs893507 (*TCERG1L*) in a cohort of this size can be accounted for by the observation that toxicity-associated pharmacogenomic variants tend to have a larger effect size^22^. Nevertheless, establishing worldwide collaborations to perform future studies with even larger sample sizes remains the ideal strategy to discover additional genome-wide significant variants that predict adverse drug reactions in childhood cancer patients. In the discovery cohort and second replication cohort, we were unable to perform a robust assessment of ototoxic co-medications and their potential influence on the strength of the *TCERG1L* genetic association because retrospective patient data did not capture complete details of these co-medications. Furthermore, eQTL evidence for the effect of rs893507 on gene-expression levels was not found, possibly due to limited sample sizes in these tissues. However, eQTLs are highly tissue-specific (and often even situationally specific) thus the absence of an eQTL between rs893507 and *TCERG1L* remains equivocal. In addition to a genetic association, we were able to functionally implicate *TCERG1L* in the development of cisplatin-induced inflammatory response and toxicity. We observed an inverse correlation of *TCERG1L* with cellular responses to cisplatin suggesting a plausible biological link between our association results and the mechanism of cisplatin-related hearing loss in non-cranial-irradiated subjects. The functional data are consistent with rs893507 having a deleterious effect on *TCERG1L* function.

In conclusion, the combined results of this study suggest that cisplatin-treated, non-cranial irradiated childhood cancer patients with a genetic intronic variant (rs893507) in *TCERG1L* have a 3.11 fold increased odds of developing cisplatin-induced hearing loss. We found evidence that *TCERG1L* is related to direct cisplatin-induced hearing loss in childhood cancer patients, the results of which were strengthened through replication in two independent replication cohorts, and biological validation in vitro. Our study shows statistical and functional evidence for the involvement of *TCERG1L* in cisplatin-induced inflammatory response and toxicity. Even though cochlear inflammation induced by cisplatin can lead to inner ear damage and hearing loss^23^, future studies are needed to further validate the functional impact of the variant related to hearing loss, preferably by use of human cochlear sections, and to determine the additional (epi-)genetic regulation of the genetic variant associated with cisplatin-induced hearing loss.

## Methods

### Patients and treatment

The discovery cohort (PCL discovery cohort, D) consists of childhood cancer patients from the PCL consortium, a multicenter cohort of childhood cancer patients and survivors across Europe designed to address ototoxicity, fertility impairment, and quality of life. In the current study, 390 cisplatin-treated, non-cranial irradiated patients were included. Subjects were diagnosed and treated for childhood cancer in Europe. A detailed description of the PCL discovery cohort is available^24^. Inclusion criteria for this study were patients: (1) diagnosed with cancer before the age of 19 years; (2) initially treated with cisplatin, as a single platinum drug during childhood cancer treatment, or switched from cisplatin to carboplatin during treatment; (3) did not receive cranial or inner ear radiation; (4) completed their chemotherapy treatment; (5) had at least one pure tone audiometric evaluation available within two years after completion of chemotherapy; (6) had their biomaterial (blood or saliva) available for DNA extraction; and (7) no baseline hearing loss. Patients with hearing loss before the start of chemotherapy and patients with initial treatment of carboplatin were excluded. Patient data (including demographic, diagnostic, audiological, and treatment-related information) was collected retrospectively from medical records at participating institutions in Europe. Patients were enrolled after approval had been obtained from local review boards and written informed consent was obtained from patients, parents, or legal guardians. The PCL study was approved by the local ethics committees: Kantonale Ethikkommission Bern, 362/2015; Comitate Etico Regionale, 507REG2014; Ethical Committee University Hospital Brno, June 11, 2016; Ethics Committee Fakultni Nemocnice v Motole Prague, EK-1447/14; De Videnskabsetiske Komiteer Region Hovedstaden, H-1-2014-125; Ethikkommission Medizinische Universität Graz, 27-015 ex 14/15; Ethikkommission der Universität Ulm, 160/17; Ethikkommission der Universität zu Lübeck, 14/181; Ethik-Kommission der Ärztekammer Westfalen-Lippe und der Westfälischen Wilhelms-Universität Münster, 2014-619; Medische Ethische Toetsings Commissie Erasmus MC, MEC-2014-633; Medisch Ethische Toetsingscommissie, 2015_202 (for centers in the Netherlands which included the Erasmus Medical Center, Academic Medical Center, University Medical Center Groningen, and Princess Máxima Center for Pediatric Oncology)^9^.

The first replication cohort (Canadian replication cohort, R1) consisted of childhood cancer patients (*n* = 192) treated with cisplatin from the Canadian Pharmacogenomics Network for Drug Safety (CPNDS), a national research and patients care network established to reduce serious adverse drug reactions in children (Vancouver, BC, Canada)^25^ (http://cpnds.ubc.ca/). Participants in this replication cohort fulfilled all inclusion criteria of this study as described above. In addition, cases of cisplatin-induced hearing loss have a bilateral hearing loss only, and unilateral hearing loss cases with normal hearing in the contralateral ear are not considered cisplatin-induced. Demographic, diagnostic, audiological, and treatment-related data were retrieved from medical records. The study was approved by the research ethics board of each of the participating institutions of the enrolled patients and written informed consent was obtained from each patient or their legal guardian^9^.

An independent second replication cohort (PCL second replication cohort, R2) consisted of childhood cancer survivors from the PCL cohort. Participants were enrolled both retrospectively and prospectively (i.e., chemotherapy was started and finished during the 5-year term of PCL). Eligibility criteria were: (1) no participation in the discovery PCL cohort (D); (2) age at diagnosis <19 years; (3) treatment with cisplatin or cisplatin and carboplatin; and (4) at least one pure tone audiometric evaluation available within 5 years after the end of chemotherapy. Exclusion criteria were (1) non-consent; (2) hearing loss before the start of platinum treatment; and (3) cranial irradiation. Identified case-patients (i.e., hearing loss grade ≥2b according to Muenster Classification) were matched 1:1 with controls (i.e., patients with Muenster grade 0–2a) for sex, age at diagnosis (tolerance 5 years), and cumulative cisplatin dose (tolerance 50 mg/m^2^). Patients were enrolled after approval was obtained from local review boards and written informed consent was obtained from patients, parents, or legal guardians^9^.

A lookup of the GWAS hits was performed in the discovery cohort of the Cohorts for Heart and Aging Research in Genomic Epidemiology (CHARGE) to evaluate a possible association between the SNP and age-related hearing loss. This cohort on age-related hearing impairment consisted of 9,675 subjects from the general population who were all 45 years or older at the time of the study. Phenotypes were examined that represented low/mid (0.5, 1, and 2 kHz) and high-frequency hearing loss (at 4 and 8 kHz) based on pure tone audiograms.

### Hearing loss assessment and classification

The main endpoint of this study was hearing loss following cisplatin treatment. Pure tone audiometry (up to 8000 Hz) was performed in all patients after the end of platinum treatment. For all cohorts, the results of audiological examinations were graded according to Muenster classification (Supplementary Table 2)^11^. Audiogram assessors were blinded to patient characteristics, treatment factors, and genetic data. Grading was based on the worst ear, determined at the first measurement available after the end of treatment. In the current study, patients who had deleterious hearing loss grade ≥2b according to Muenster were considered cases. Patients with Muenster grade 0−2a were assigned to the comparison group. A comparison between subjects with Muenster grade 0 and subjects with Muenster grade ≥2b was not adequately powered due to a lack of sufficient grade 0 subjects.

### Genotyping and quality control

In the PCL discovery cohort, DNA was isolated from blood and saliva samples. Blood samples were stored at ≤−20 °C and shipped on dry ice, and saliva was stored and shipped at room temperature. The salting-out method was applied to extract genomic DNA. The Illumina Infinium^©^ Global Screening Array (Illumina, San Diego, CA, USA) was used for DNA genotyping. A stringent quality control protocol containing multiple filters was applied to clean the genetic data (Supplementary Fig. 4). To remove poorly genotyped SNPs and individuals from the data, an SNP and individual call rate filter of 97.5% was employed. The Hardy–Weinberg equilibrium (HWE) test with a significance level of ${P}\,<\,{1.0}\, \ast \,{10}^{-7}$ was used to take out variants containing potential genotyping errors. Samples with extreme heterozygosity, gender mismatches, and familial relationships were removed. Thirty-two samples (8%) of non-European ancestry, identified by genetic profile, were included. To account for genetic ancestry, four principal components were calculated using PLINK (version 1.90). Imputations to Haplotype Reference Consortium (HRC r1.1) (http://www.haplotype-reference-consortium.org/site) were performed using the Michigan Imputation Server with default settings (https://imputationserver.sph.umich.edu/index.html).

Within the CPNDS cohort, all blood and saliva samples were genotyped by use of the Illumina Infinium^©^ OmniExpress array. Genotyped variants underwent stringent quality control procedures, for which the QCTOOL (version 2), GTOOL (version 0.7.5), and PLINK (version 1.90) were used. Genetic variants with a call rate of <95%, a minor allele frequency of <1% in both cases and controls, and variants deviating from HWE genotype distributions (significance level ${P}\, < \,{1.0}\, \ast \,{10}^{-6}$) were removed. After harmonization of the genotype data using Genotype Harmonizer^26^, principal component analyses were performed using EIGENSOFT (version 5)^27,28^. Sixty samples (31%) of non-European ancestry were included. Imputation was performed using SHAPEIT2^29^ and IMPUTE2^30^, using the Phase 3 1000 Genomes Project samples as a reference (https://www.internationalgenome.org/category/phase-3/).

In the PCL replication cohort, genotyping calls of variants replicating in the Canadian replication cohort were validated by using TaqMan^®^ PCR^31^.

### GWAS and replication

Measurements were taken from distinct samples. For the GWAS analyses, we used logistic regression models including age at diagnosis (linear term), sex, total cumulative cisplatin dose (linear term), and four principal components using rvtests (Supplementary Table 7). Due to the limited sample size, we a priori determined that genome-wide significant findings (*P* < 5 × 10^−8^) were unlikely. We therefore decided to pursue variants with suggestive levels of association (*P* < 1 × 10^−5^) for replication.

To estimate potential cisplatin dose–response effects, we first analyzed the association between deleterious hearing loss and cisplatin dose continuously, as well as stratified in groups of <360, 360–480, and >480 mg/m^2^. Next, a potential cisplatin dose–response effect with the genetic variant was estimated by modeling cisplatin dose as a continuous variable as well as categorical and ran a logistic regression model including interaction terms for the genetic variant and cisplatin dose.

Functional SNP annotations were applied by the FUMA web application, and gene analysis and gene-set analysis were performed by MAGMA v1.6, integrated into FUMA^32^.

For replication, firstly, the Canadian childhood cohort GWAS data were used^25^. Secondly, a candidate SNP approach was used in the PCL adult childhood cancer cohort of non-cranial-irradiated childhood cancer survivors. Variants that were prioritized in the discovery analyses were extracted from the Canadian first replication cohort genotype data. These variants were examined for evidence of replication using logistic regression, adjusted for age at diagnosis, vincristine, germ cell tumor type, and principal components 1–4, using SNPTEST. Adjusted ORs and 95% CIs (two-sided) were calculated using the R package PredictABEL. *P* < 0.01 (0.05/7 gene variants, correcting for multiple testing by Bonferroni correction) were considered statistically significant. Only variants replicating in the Canadian first replication cohort were candidates for replication in the PCL second replication cohort. Association of the variant with cisplatin-induced hearing loss was examined using logistic regression, assuming an additive effect of the minor allele. Adjustments were made for sex, age at diagnosis, and cumulative cisplatin dose. Data from the discovery and replication cohorts were combined and examined using meta-analytic approaches in R version 3.5.1, package “rmeta”^33^. An inverse-variance meta-analysis was used.

### Experimental validation

Functional validation experiments were performed to determine the effect of knockdown or overexpression of *TCERG1L* on cisplatin cytotoxicity and cisplatin-induced inflammatory cytokine production. We used HeLa cells as a model of human cultured cells (ATCC catalog no. CCL-2). To determine the impact of gene knockdown on general cell viability in the presence and absence of varying concentrations of cisplatin in HeLa cells, MTT (3-(4,5-dimethylthiazol-2-yl)-2,5-diphenyltetrazolium bromide) cell viability assays were used. The cisplatin-induced inflammatory response was assessed by interleukin-8 (IL-8) enzyme-linked immunosorbent assays (Thermofisher). Knockdown experiments were all performed using pre-designed *TCERG1L* siRNA (hs.Ri.TCERG1L.13.2; Integrated DNA Technologies) according to the manufacturer’s specifications. siRNA transfection was performed with Lipofectamine RNAiMAX according to the manufacturer’s protocol (Thermofisher). Real-time quantitative PCR (RT-qPCR), using *TCERG1L* specific primer-probe mix (Hs.PT.58.40562685; Integrated DNA Technologies) showed 70% silencing efficiency of *TCERG1L* compared to the non-targeting siRNA control. Overexpression experiments were all performed using ectopic transfection of pCMV6*TCERG1L* (RC207369; OriGene) using JetPrime reagent (PolyPlus) according to the manufacturer’s instructions.

For *TCERG1L* overexpression experiments cells were first seeded at 1.5–3 × 10^6^ cells in 10 cm dishes for 24 h prior to transfection. Transfected cells were then trypsinisized and seeded into either 96-well plates, with 5000 cells per well, or 24-well plates, with 70,000 cells per well. Newly transferred cells were allowed to grow for another 24 h prior to cisplatin treatment. Treatment with cisplatin proceeded for another 48 h prior to supernatant collection and cell viability assays. For *TCERG1L* silencing experiments, cells were treated as above or seeded directly into 24-well plates prior to transfection. Cell viability curves were generated in Prism7 using non-linear curve fits normalized response and compared using Extra sum-of-squares *F*-test. Relative IL-8 secretion was compared using a two-tailed Student’s *t*-test.

For the expression changes of *TCERG1L* and IL-6 induced by cisplatin treatment across various mouse tissues, we made use of a publicly available dataset at the NCBI gene expression omnibus (GEO) under accession number GSE117167^34^. This dataset comprises total RNA from liver, kidney, spleen, and lung tissue specimens from 6-month-old female mice in a pure C57Bl6/J genetic background, which were collected after 4 h following cisplatin (10 mg/kg) or saline IP injection and sequenced using the HiSeq 4000 Illumina RNA-seq platform^35^. The raw data files were downloaded and subjected to our in-house generated data analysis pipeline. Sequence adaptors were removed from the sequence reads using Trimmomatic version 0.39 and the trimmed reads were subsequently aligned to the mouse genome using Star version 2.7.0f with gencode.vM20.annotation.gtf and GRCm38. p6.genome.fa as annotation and genome (http://gencodegenes.org/mouse/release_M20.html). Read counts for each gene were obtained using FeatureCounts and log-fold changes and false discovery rates were quantified using EdgeR version 3.24.3. Visualization of the relative fold changes (cisplatin versus saline) across the various organs was performed in GraphPad Prism version 8.4.2 (GraphPad Software, La Jolla, CA, USA).

### Reporting summary

Further information on research design is available in the Nature Research Reporting Summary linked to this article.

## Supplementary information

Supplementary Information

Reporting Summary

## Data Availability

The data generated and analyzed during this study are described in the following data record: 10.6084/m9.figshare.14260988^9^. Summary statistics from GWAS analyses of participant blood and saliva samples (Meijer_01032021_cisplatin_induced_hearing_loss.tsv) are openly available from the GWAS Catalog: GCST90013831^12^. Gene expression profiling data from mouse tissues in response to cisplatin treatment, are openly available from Gene Expression Omnibus: https://identifiers.org/geo:GSE117167^34^. Data supporting Supplementary Table 1, Supplementary Tables 4–7 and Supplementary Fig. 3 cannot be made openly available due to restrictions based on privacy regulations and informed consent of the participants in Europe and Canada. Requests for data access should be sent to MvdHE (m.m.vandenheuvel-eibrink@prinsesmaximacentrum.nl) for the PCL discovery cohort, BC (bcarleton@popi.ubc.ca) for the Canadian replication cohort, OZ for the (Oliver.Zolk@mhb-fontane.de) PCL replication cohort and AN (a.nagtegaal@erasmusmc.nl) for the CHARGE cohort.

## Code availability

Only standard scripts were used for this study. No custom codes or mathematical algorithms were applied.
