# Supplementary material for: TCERG1L allelic variation is associated with cisplatin-induced hearing loss in childhood cancer, a PanCareLIFE study
Source: NPJ Precis Oncol. 2021 Jul 14;5:64. doi: 10.1038/s41698-021-00178-z (PMC8280110; doi:10.1038/s41698-021-00178-z)
Supplement: Supplementary file 1 — Supplementary Information [file 41698_2021_178_MOESM1_ESM.pdf]

Supplementary Table 1. Clinical characteristics of the PCL discovery cohort, Canadian first replication cohort and PCL second replication cohort.

	PCL discovery cohort (D): N=390			Canadian replication cohort (R1): N=192			PCL second replication cohort (R2): N=188*		
	Muenster grade ≥2b (n=168)	Muenster grade <2b (n=222)	P	Muenster grade ≥2b (n=115)	Muenster grade <2b (n=77)	P	Muenster grade ≥2b (n=94)	Muenster grade <2b (n=94)	P
Median age at diagnosis, years (range)	9.5 (0.0-18.8)	12.8 (0.1-18.7)	0.001	3.0 (2.0-8.0)	9.0 (2.0-13.0)	0.002	10.5 (0.8-17.6)	13.10 (0.3-18.0)	0.07
Calendar year of diagnosis, n (%)			0.009			0.51			NA
1980-1994	16 (9.5)	9 (4.1)		16 (13.9)	13 (16.9)		2 (2.1)	3 (3.2)	
1995-2005	70 (41.7)	74 (33.3)		65 (56.5)	37 (48.1)		43 (45.7)	25 (26.6)	
2006-2016	82 (48.8)	139 (62.6)		34 (29.6)	27 (35.1)		49 (52.1)	66 (70.2)	
Median time to follow-up, years (range)	0.4 (0.0-3.0)	0.3 (0.0-2.5)	0.001	0.7 (0.2-11.4)	0.8 (0.1-16.2)	0.91	1.6 (0.0-17.2)	1.7 (0-11.8)	0.77
Median age at follow-up, years (range)	9.9 (0.4-19.4)	13.1 (0.3-19.7)	0.001	5.0 (0.5-16.5)	10.9 (0.9-19.6)	0.001	14.9 (2.5-29.4)	16.3 (1.5-26.7)	0.38
Median TCD cisplatin, mg/m^2 (range)	480 (83-808)	453 (40-950)	0.005	400 (345-480)	400 (300-480)	0.55	480 (83-730)	480 (120-770)	0.90
Male sex, n (%)	79 (47)	102 (45.9)	0.59	68 (59.1)	40 (51.9)	0.37	48 (51.1)	48 (51.1)	1.00
Tumor type, n (%)			NA			NA			NA
Carcinoma	2 (1.2)	6 (2.7)		0 (0)	3 (3.9)		0 (0)	0 (0)	
CNS tumor	3 (1.8)	2 (0.9)		6 (5.2)	3 (3.9)		5 (5.3)	4 (4.3)	
Germ cell tumor	12 (7.1)	51 (23.0)		8 (7.0)	19 (24.7)		2 (2.1)	16 (17.0)	
Hepatoblastoma	13 (7.7)	20 (9.0)		29 (25.2)	10 (13.0)		5 (5.3)	5 (5.3)	
Hodgkin lymphoma	1 (0.6)	2 (0.9)		0 (0)	0 (0)		0 (0)	0 (0)	
Neuroblastoma	44 (26.2)	18 (8.1)		42 (36.5)	15 (19.5)		6 (6.4)	3 (3.2)	
Osteosarcoma	90 (53.6)	118 (53.2)		28 (24.3)	21 (27.3)		58 (61.7)	53 (56.4)	
(Soft tissue) sarcoma	2 (1.2)	1 (0.5)		0 (0)	2 (2.6)		0 (0)	0 (0)	
Other bone tumor	0 (0.0)	1 (0.5)		0 (0)	0 (0)		0 (0)	0 (0)	
Other	1 (0.6)	0 (0.0)		2 (1.8)	4 (5.1)		14 (14.9)	0 (0)	
Missing	0 (0.0)	3 (1.4)		0 (0)	0 (0)		4 (4.3)	9 (9.6)	
Additional carboplatin, n (%)	59 (35.1)	17 (7.6)	<0.001	49 (42.6)	12 (15.6)	$7x10^{-5}$	38 (40.4)	15 (16.0)	$3x10^{-4}$

* This replication cohort represents the non-cranial irradiated cisplatin treated subset of survivors of childhood cancer included in PCL. The cases and controls were matched 1:1 based on gender, age at diagnosis and total cumulative dose cisplatin.

P-values represent statistical differences in clinical characteristics between patients with deleterious hearing loss, and patients without hearing loss.

Abbreviations: NA = not applicable as N is less than 5; PCL = PanCareLIFE; TCD = total cumulative dose.

Supplementary Table 2. The Muenster classification for ototoxicity grading.

Grade	Muenster classification	
0	≤10 dB HL at all frequencies	Non-deleterious hearing loss
1	>10 and ≤20 dB HL in at least one frequency, or tinnitus	
2a	>20 – ≤40 dB HL at ≥4 kHz	
2b	>40 – ≤ 60 dB HL at ≥4 kHz	Deleterious hearing loss
2c	>60 dB HL at ≥4 kHz	
3a	>20 – ≤40 dB HL at <4 kHz	
3b	>40 – ≤60 dB HL at <4 kHz	
3c	>60 – <80 dB HL at <4 kHz	
4	≥80 dB HL at <4 kHz	

Abbreviations: dB = decibel; HL = hearing loss; kHz = kilohertz.

Supplementary Table 3. Genetic analyses of the PCL discovery cohort, Canadian first replication cohort and PCL second replication cohort.

	PCL discovery cohort (n=390)	Canadian first replication cohort (n=192)	PCL second replication cohort (n=188)
Cohort	Discovery cohort	First replication cohort	Second replication cohort
DNA samples	Blood or saliva	Blood or saliva	Blood or saliva
Genotyping	Infinium© Global Screening Array	Illumina© Infinium OmniExpress Array	Taqman PCR
Quality control program	PLINK v1.9	PLINK v1.9	NA
Variant call rate	<97.5%	<95%	NA
Hardy-Weinberg equilibrium	$P<1x10^{-7}$	$P<1x10^{-6}$	NA
PCA analysis	PLINK v1.9	EIGENSOFT v5	NA
Non-CEU samples removed	No	No	NA
Imputation method	Michigan Imputation Server	SHAPEIT v2, IMPUTE2 v2.3.2	NA
Reference panel	Haplotype Reference Consortium	Phase 3 1000 Genomes Project	NA

Abbreviations: CEU = European descent; NA: not applicable; PCA = principal component analysis; PCL = PanCareLIFE.

Supplementary Table 4. Genotype frequencies and association results for heterozygote and homozygote TCERG1L carriers in the discovery cohort and replication cohorts.

Study (N)	Genotype (N)	OR (95% CI)	P-value
Discovery (390)	TT (274)	1	REF
	TC (108)	2.57 (1.63-4.06)	<0.001
	CC (8)	2.95 (0.69-12.59)	0.15
R1 (192)	TT (159)	1	REF
	TC (32)	1.91 (0.83-4.39)	0.13
	CC (1)	2.25 (0.09-55.98)	0.62
R2 (188)	TT (150)	1	REF
	TC (36)	6.90 (2.71-17.58)	<0.001
	CC (2)	1.38 (0.08-22.50	0.82
Combined analysis (770)	TT (583)	1	REF
	TC (176)	2.55 (1.79-3.62)	<0.001
	CC (11)	1.84 (0.53-6.34)	0.18

Abbreviations: OR=odds ratio; R1=replication cohort 1; R2=replication cohort 2.

Supplementary Table 5. Look up results of rs893507 in the CHARGE cohort hearing impairment.

Phenotype	rsID	Ref/Eff	EAF	Beta	SE	P-value	N
HIGH	rs893507	T/C	0.15	-0.03	0.02	0.2	9,675
LOW	rs893507	T/C	0.15	-0.02	0.02	0.4	9,675

The rs893507 variant we found in our discovery cohort did not show significant replication in the CHARGE cohort hearing impairment.

Abbreviations: EAF = effect allele frequency; Eff = effect allele; HIGH = High frequency hearing loss (4 and 8 kHz); LOW = Low and mid frequency hearing loss (0.5, 1 & 2 kHz); Ref = reference allele; SE = standard error.

Supplementary Table 6. Association between *TCERG1L* and deleterious hearing loss in the PCL second replication cohort, for childhood cancer survivors treated with and without cranial irradiation.

	PCL second replication cohort		
	Analysis in matched* non-cranial irradiated survivors (N=188)	Analysis in cranial irradiated survivors (N=553)	Total (N=741)
Muenster grade ≥2b	94	92	186
Muenster grade <2b	94	461	555
TCERG1L rs893507	OR 5.45 (95% CI 2.3-12.8)	OR 1.25 (95% CI 0.6-2.5)	OR 1.32 (95% CI 0.9-1.9)

*Survivors were matched for age, cumulative dose cisplatin and gender.

Supplementary Table 7. Risk factors associated with hearing loss in the discovery cohort, results of univariate and multivariate logistic regression analyses.

	Muenster ≥2b N=168 (43%)	Muenster <2b N=222 (57%)	UVA OR (95% CI)	MVA OR (95% CI)
Age at diagnosis per 5 years increase, median (range)	9.5 (0.0-18.8)	12.8 (0.1-19.3)	0.74 (0.56-0.92)*	0.76 (0.55-0.96)*
Sex				
Female	66 (46%)	97 (49%)	REF	REF
Male	79 (54%)	102 (51%)	0.88 (0.57-1.35)	0.78 (0.50-1.22)
TCD cisplatin per 100 mg/m^2 increase, median (range)	480 (83-808)	453 (40-950)	1.35 (1.15-1.55)*	1.35 (1.15-1.55)*

*P<0.05; Abbreviations: MVA=multivariate analysis; TCD=total cumulative dose; UVA=univariate analysis.

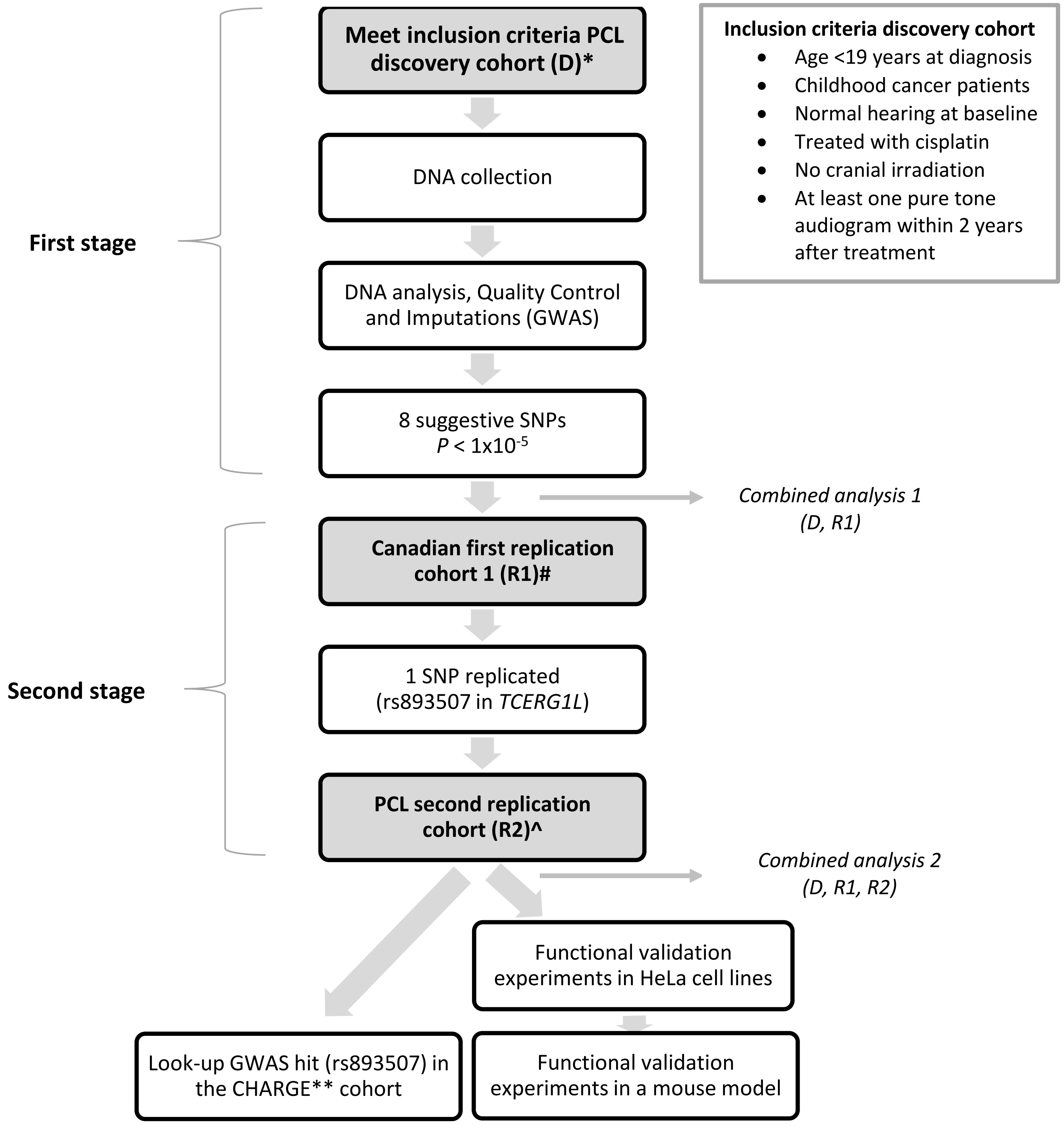

Supplementary Figure 1. Flowchart of the two-stage design of the discovery logistics, replication process, functional validation, and look-up of results in a healthy population.

* The PCL discovery cohort (D) represent 390 cisplatin treated non-cranial irradiated childhood cancer patients (median age at diagnoses 11.1 years, 43.0% developed Muenster ≥2b hearing loss). # The independent Canadian first replication cohort (R1) represent 192 non-cranial irradiated childhood cancer patients (median age at diagnosis 4.1 years, 59.9% developed Muenster ≥2b hearing loss).^ The independent PCL second replication cohort (R2) represent 188 childhood cancer survivors (median age at diagnosis 11.1 years, 50.0% developed Muenster ≥2b hearing loss).

** CHARGE age-related hearing impairment cohort (9,675 subjects of the general population, age >45 years at time of study).

Abbreviations: CHARGE = Cohorts for Heart and Aging Research in Genomic Epidemiology; DNA = deoxyribonucleic acid; GWAS = genome wide association study; PCL = PanCareLIFE; SNP = single nucleotide polymorphism; TCERG1L = Transcription Elongation Regulator 1-Like.

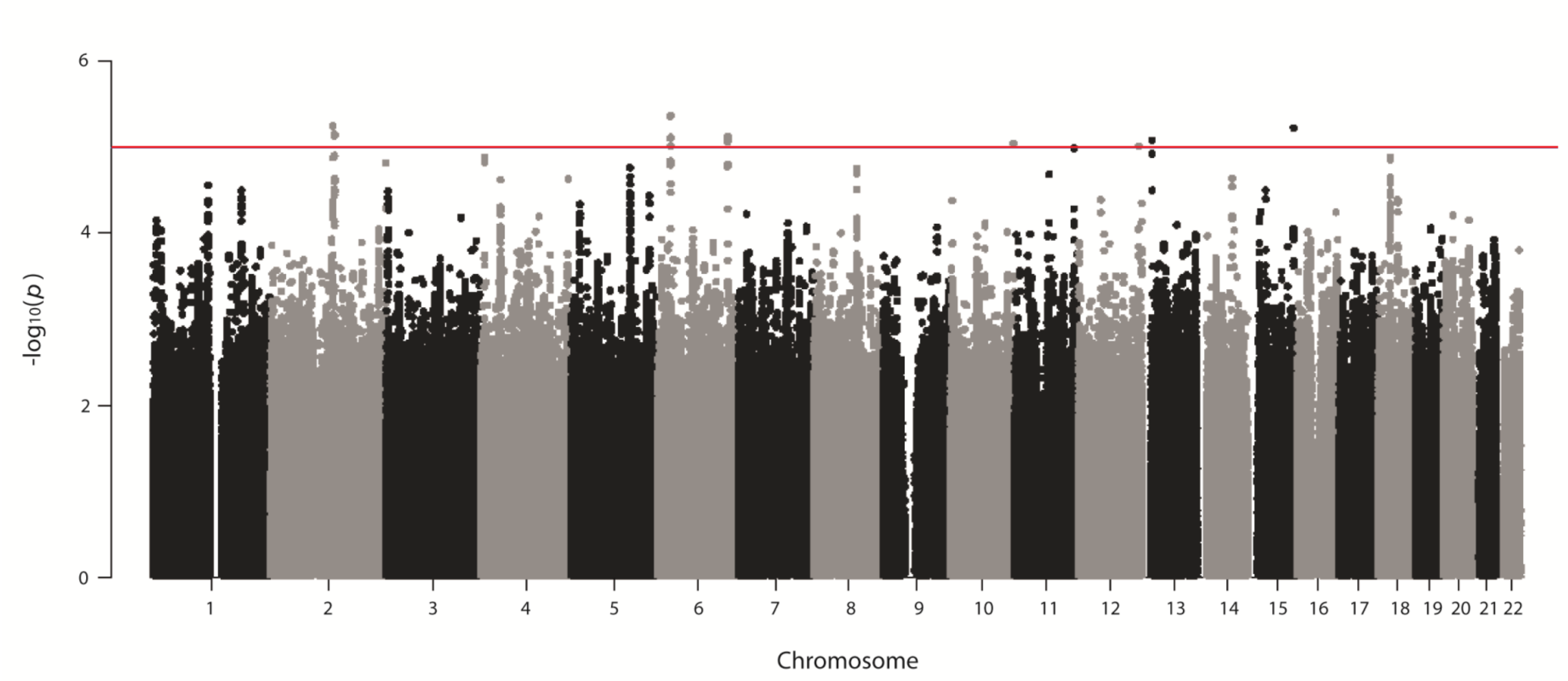

Supplementary Figure 2. Manhattan plot showing the top SNPs identified by GWAS in the PCL discovery cohort. The suggestive value for association (P=1.0×10^{-5}) is presented by the red line.

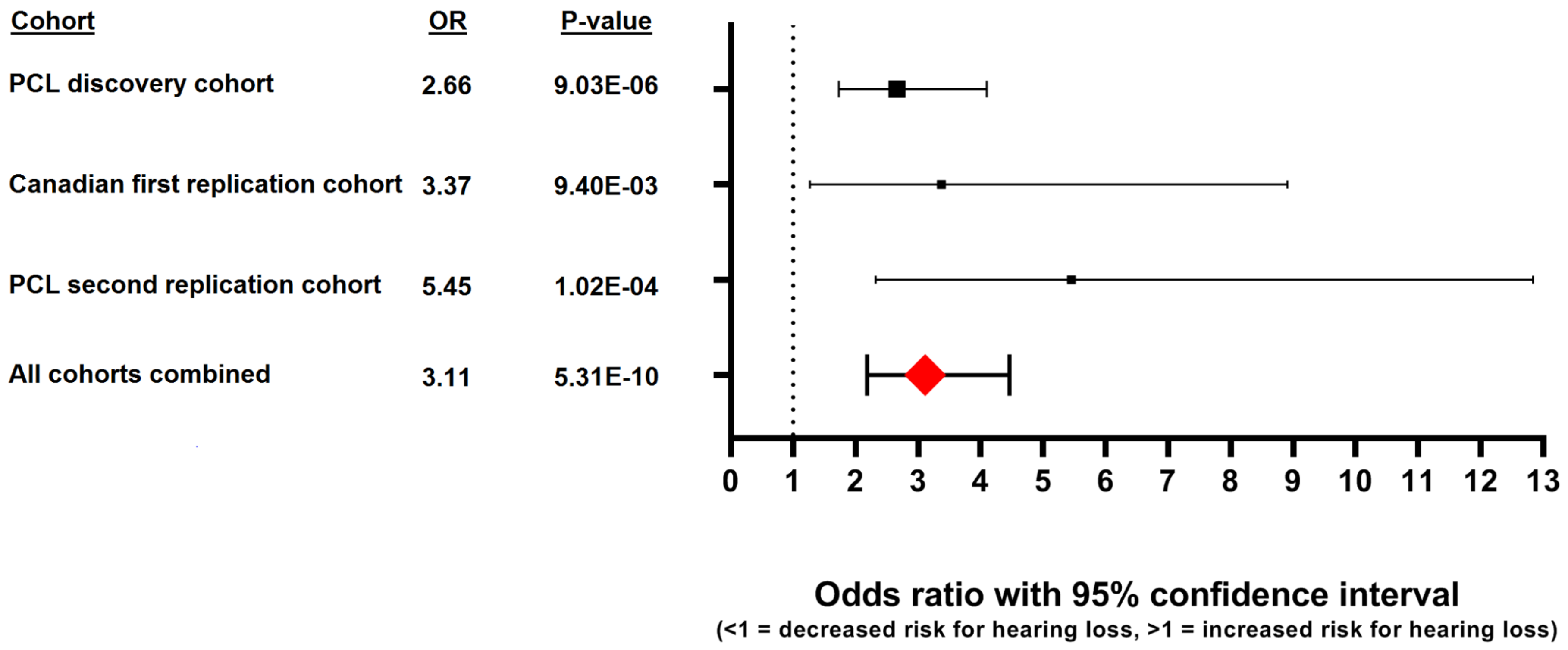

Supplementary Figure 3. Variation in TCERG1L (rs893507) association with cisplatin-induced hearing loss is genome-wide significant by combined analysis in three independent patient cohorts (I^2 = 8.1). The combined analysis showed that patients who carry the C allele increased the odds of cisplatin-induced hearing loss with 3.11 (95% CI 2.2-4.5) compared to patients who carry the T allele. The forest plot was created in GraphPad Prism version 8.3.0.

Abbreviations: OR = odds ratio; PCL = PanCareLIFE.

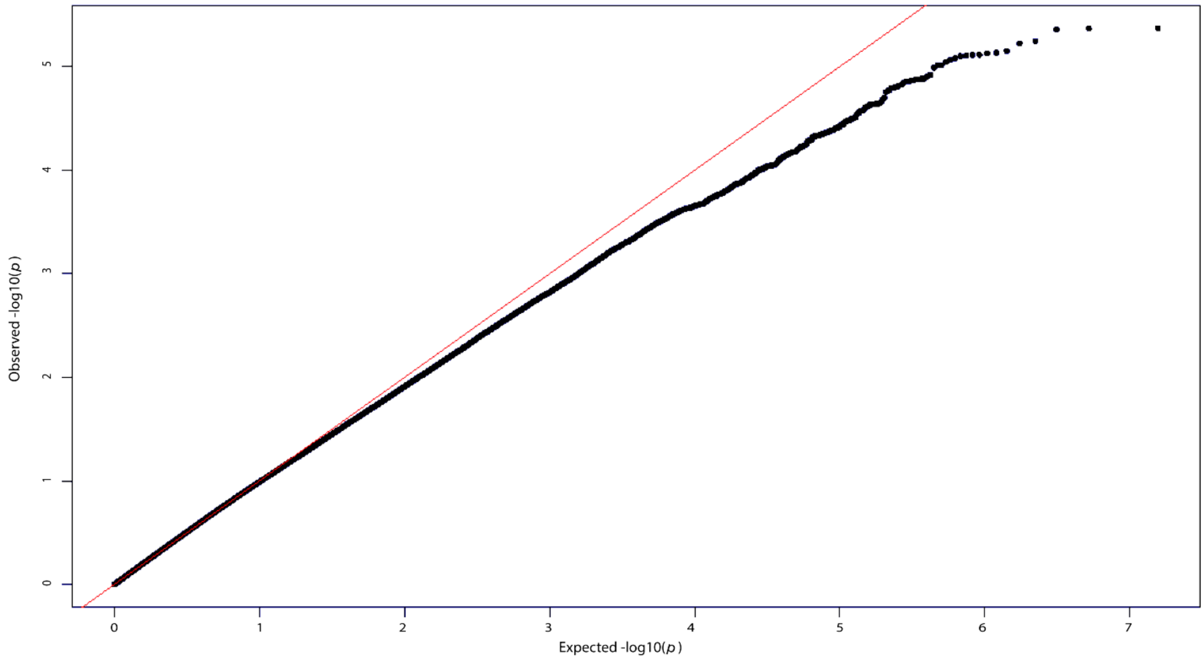

Supplementary Figure 4. Quantile-quantile (QQ) plot for genome-wide association with cisplatin-induced hearing loss (λ = 1.02).
