# Supplementary material for: TCERG1L allelic variation is associated with cisplatin-induced hearing loss in childhood cancer, a PanCareLIFE study
Source: NPJ Precis Oncol. 2021 Jul 14;5:64. doi: 10.1038/s41698-021-00178-z (PMC8280110; doi:10.1038/s41698-021-00178-z)
Supplement: Supplementary file 2 — Reporting Summary [file 41698_2021_178_MOESM2_ESM.pdf]

nature research

Corresponding author(s): AJM Meijer

Last updated by author(s): Jan 28, 2021

Reporting Summary

Nature Research wishes to improve the reproducibility of the work that we publish. This form provides structure for consistency and transparency in reporting. For further information on Nature Research policies, see our Editorial Policies and the Editorial Policy Checklist.

Statistics

For all statistical analyses, confirm that the following items are present in the figure legend, table legend, main text, or Methods section.

n/a	Confirmed	
☐	☒	The exact sample size (n) for each experimental group/condition, given as a discrete number and unit of measurement
☐	☒	A statement on whether measurements were taken from distinct samples or whether the same sample was measured repeatedly
☐	☒	The statistical test(s) used AND whether they are one- or two-sided<br>*Only common tests should be described solely by name; describe more complex techniques in the Methods section.*
☐	☒	A description of all covariates tested
☐	☒	A description of any assumptions or corrections, such as tests of normality and adjustment for multiple comparisons
☐	☒	A full description of the statistical parameters including central tendency (e.g. means) or other basic estimates (e.g. regression coefficient) AND variation (e.g. standard deviation) or associated estimates of uncertainty (e.g. confidence intervals)
☐	☒	For null hypothesis testing, the test statistic (e.g. F, t, r) with confidence intervals, effect sizes, degrees of freedom and P value noted<br>*Give P values as exact values whenever suitable.*
☒	☐	For Bayesian analysis, information on the choice of priors and Markov chain Monte Carlo settings
☒	☐	For hierarchical and complex designs, identification of the appropriate level for tests and full reporting of outcomes
☐	☒	Estimates of effect sizes (e.g. Cohen's d, Pearson's r), indicating how they were calculated

Our web collection on statistics for biologists contains articles on many of the points above.

Software and code

Policy information about availability of computer code

Data collection	No software was used for data collection.
Data analysis	For the PCL discovery cohort GWAS, logistic regression model were analyzed by using rvtests. In the Canadian replication cohort, variants were examined for evidence of replication by logistic regression using SNPTEST. Adjusted ORs and 95% CIs (two-sided) were calculated using the R package PredictABEL. Data from the discovery and replication cohorts were combined and examined using meta-analytic approaches in R version 3.5.1, package “rmeta”. For the functional experiments, cell viability curves were generated in Prism7 using non-linear curve fits to normalized response and compared using Extra sum-of-squares F-test. Relative IL-8 secretion was compared using a two-tailed student’s t-test.

For manuscripts utilizing custom algorithms or software that are central to the research but not yet described in published literature, software must be made available to editors and reviewers. We strongly encourage code deposition in a community repository (e.g. GitHub). See the Nature Research guidelines for submitting code & software for further information.

Data

Policy information about availability of data

All manuscripts must include a data availability statement. This statement should provide the following information, where applicable:

- Accession codes, unique identifiers, or web links for publicly available datasets
- A list of figures that have associated raw data
- A description of any restrictions on data availability

Because of restrictions based on privacy regulations and informed consent of the participants in Europe and Canada, data cannot be made freely available in a public repository. Data requests can be directed towards the PIs of the three included cohorts (MvdHE, BC, OZ). The GWAS summary statistics of the discovery cohort will be uploaded in GWAS Catalog directly after publication. The PanCareLIFE study was approved by the local ethics committees: Kantonale Ethikkommission

Bern, 362/2015; Comitate Etico Regionale, 507REG2014; Ethical Committee University Hospital Brno, June 11, 2016; Ethics Committee Fakultni Nemocnice v Motole Prague, EK-1447/14; De Videnskabsetiske Komiteer Region Hovedstaden, H-1-2014-125; Ethikkommission Medizinische Universität Graz, 27-015 ex 14/15; Ethikkommission der Universität Ulm, 160/17; Ethikkommission der Universität zu Lübeck, 14/181; Ethik-Kommission der Ärztekammer Westfalen-Lippe und der Westfälischen Wilhelms-Universität Münster, 2014-619; Medische Ethische Toetsings Commissie Erasmus MC, MEC-2014-633; Medisch Ethische Toetsingscommissie, 2015_202. CPNDS studies were approved by the Canadian Research Ethics Board (i H04-70358). All participants provided written informed consent to participate in the study and to have their information obtained from treating physicians. RNA expression data files are publicly available at the NCBI gene expression omnibus (GEO) under accession number GSE117167.

Field-specific reporting

Please select the one below that is the best fit for your research. If you are not sure, read the appropriate sections before making your selection.

☒ Life sciences ☐ Behavioural & social sciences ☐ Ecological, evolutionary & environmental sciences

For a reference copy of the document with all sections, see nature.com/documents/nr-reporting-summary-flat.pdf

Life sciences study design

All studies must disclose on these points even when the disclosure is negative.

Sample size	To estimate the number of cases required for the GWAS analyses, a sample size calculation was performed. Assuming a risk allele frequency of 0.2, a case to control ratio of 1:1, and a P value threshold of P<5*10–8 for the GWAS analysis, a cohort of 574 patients was considered sufficient to detect an odds ratio of at least 2.8 with a statistical power of 80% in the design of the study.
Data exclusions	Exclusion criteria for this study were patients: (1) diagnosed with cancer at >19 years of age; (2) not initially treated with cisplatin, or initially treated with carboplatin; (3) had received cranial or inner ear radiation; (4) had not completed their chemotherapy treatment; (5) did not have at least one pure tone audiometric evaluation available after completion of chemotherapy; (6) did not have their biomaterial (blood or saliva) available for DNA extraction; and 7) had hearing loss before start of chemotherapy.
Replication	The first replication cohort consisted of childhood cancer patients (n=192) treated with cisplatin from the Canadian Pharmacogenomics Network for Drug Safety (CPNDS). A second replication cohort consisted of a subset of childhood cancer survivors from the PCL WP5 cohort (n=188).
Randomization	The design did not include randomization. To control for covariates, we used logistic regression models including age at diagnosis, sex, total cumulative cisplatin dose and four principal components using rvtests.
Blinding	The data were stripped of all identifiers and assigned a unique PCL-ID number, rendering the data pseudonymous for the investigators of this study. Data providers sent the pseudonymized original audiograms to the audiometry center (Department of Phoniatry and Pediatric Audiology, University Hospital Muenster, Germany) for standardized review. The audiogram assessors were blinded to patient characteristics including their treatment, such as platinum compound, platinum dose, or cranial irradiation.

Reporting for specific materials, systems and methods

We require information from authors about some types of materials, experimental systems and methods used in many studies. Here, indicate whether each material, system or method listed is relevant to your study. If you are not sure if a list item applies to your research, read the appropriate section before selecting a response.

Materials & experimental systems

n/a	Involved in the study	
☒	☐	Antibodies
☐	☒	Eukaryotic cell lines
☒	☐	Palaeontology and archaeology
☒	☐	Animals and other organisms
☐	☒	Human research participants
☒	☐	Clinical data
☒	☐	Dual use research of concern

Methods

n/a	Involved in the study	
☒	☐	ChIP-seq
☒	☐	Flow cytometry
☒	☐	MRI-based neuroimaging

Eukaryotic cell lines

Policy information about cell lines

Cell line source(s)	HeLa cells were obtained from the American Type Culture Collection (ATCC).
Authentication	The cell line was not authenticated.
Mycoplasma contamination	PCR testing for mycoplasma contamination was negative.

Commonly misidentified lines
(See ICLAC register)

HeLa cells were the only cell line used in this study.

Human research participants

Policy information about studies involving human research participants

Population characteristics	PCL discovery cohort (n=390): median age at diagnosis 11.1 years (0.0-18.8); median age at audiological testing 11.8 years (0.3-19.0); median total cumulative dose cisplatin 480 mg/m2 (range: 40-950 mg/m2). Seventy-six (19.5%) patients had been treated with additional carboplatin. One hundred sixty-eight (43.1%) patients developed Muenster ≥2b hearing loss.<br>Canadian first replication cohort (n=192): median age at diagnosis 4.1 years (0.1-18.8); median cumulative dose cisplatin 400 mg/m2 (300-480 mg/m2). One hundred fifteen patients (59.9%) developed Muenster ≥2b hearing loss.<br>PCL second replication cohort (n=188): median age at diagnosis 11.1 years (0.3-18.0); median cumulative dose of cisplatin 480 mg/m2 (83-770); 94 survivors (50%) developed Muenster ≥2b hearing loss.
Recruitment	PCL discovery cohort and PCL second replication cohort: Study participants were recruited through the PCL network consisting of 14 institutions from 7 countries: Switzerland, Italy, Czech Republic, Denmark, Germany, Austria, and the Netherlands.<br>Canadian children replication cohort: participants were recruited via the Canadian Pharmacogenomics Network for Drug Safety (CPNDS), a national research and patients care network established to reduce serious adverse drug reactions in children.<br>Data for this study was collected prospectively as well as retrospectively. Selection bias could have occurred in the retrospective collection of data. As a result of missing or unclassifiable audiograms, some patients could not be included due to a missing phenotype. Because many of the patients with missing audiograms might have had good hearing function, they might therefore no longer had follow-up(s) for audiometric testing, As a consequence, the odds of ototoxicity based on the results of this study could have been slightly overestimated.
Ethics oversight	The PanCareLIFE study was approved by the local ethics committees: Kantonale Ethikkommission Bern, 362/2015; Comitate Etico Regionale, 507REG2014; Ethical Committee University Hospital Brno, June 11, 2016; Ethics Committee Fakultni Nemocnice v Motole Prague, EK-1447/14; De Videnskabsetiske Komiteer Region Hovedstaden, H-1-2014-125; Ethikkommission Medizinische Universität Graz, 27-015 ex 14/15; Ethikkommission der Universität Ulm, 160/17; Ethikkommission der Universität zu Lübeck, 14/181; Ethik-Kommission der Ärztekammer Westfalen-Lippe und der Westfälischen Wilhelms-Universität Münster, 2014-619; Medische Ethische Toetsings Commissie Erasmus MC, MEC-2014-633; Medisch Ethische Toetsingscommissie, 2015_202. CPNDS studies were approved by the Canadian Research Ethics Board (i H04-70358). All participants provided written informed consent to participate in the study and to have their information obtained from treating physicians.

Note that full information on the approval of the study protocol must also be provided in the manuscript.
